# Rare virilizing tumor: ovarian steroid cell tumor, not otherwise specified: a case report

**DOI:** 10.1186/s13256-022-03697-w

**Published:** 2022-12-24

**Authors:** Miry Lobaton-Ginsberg, Luz María Malanco-Hernández, Aldo Ferreira-Hermosillo

**Affiliations:** 1 Universidad Anáhuac México, Campus Norte, Mexico City, Mexico; 2 Centro Oncológico Estatal ISSEMyM, Instituto de Seguridad Social del Estado de México y Municipios, Toluca, Mexico; 3 Unidad de Investigación Médica en Enfermedades Endocrinas, Hospital de Especialidades, Centro Médico Nacional Siglo XXI, Instituto Mexicano del Seguro Social, Mexico City, Mexico

**Keywords:** Ovarian neoplasms, Virilism, Testosterone, Case report

## Abstract

**Background:**

Ovarian steroid cell tumors, not otherwise specified is a rare sex cord-stromal tumor. Almost 60% of all steroid cell tumors are categorized as not otherwise specified and represent less than 0.1% of all ovarian neoplasm. Some of them are endocrinologically active, producing virilization signs in young women. The recommended treatment is primarily surgical.

**Case presentation:**

We present the case of a 20-year-old Mexican woman with secondary amenorrhea and virilization signs. She was treated with combined oral contraceptives from 13 years old, due to a misdiagnosis of polycystic ovarian syndrome. However, 4 months after stopping medication, amenorrhea and virilization signs worsened. Biochemically, she had high serum total testosterone and free testosterone levels, and a pelvic and transvaginal ultrasound followed by a pelvic tomography scan demonstrated a right adnexal tumor. She underwent right salpingo-oophorectomy and the histopathological and immunochemistry exams confirmed the diagnosis. The patient was followed for a year after surgery and until then, her menses were regular and she had no recurrence of virilization signs.

**Conclusion:**

The purpose of this case report is to alert physicians to rule out ovarian steroid cell tumor, not otherwise specified diagnosis in young women with increased testosterone after discarding common causes such as polycystic ovarian syndrome. A multidisciplinary team including a gynecologist, endocrinologist, radiologist, and pathologist should be involved for correct diagnosis at the proper time.

## Background

Ovarian steroid cell tumors, not otherwise specified (OSCT-NOS) is a rare type of sex cord-stromal tumor, representing 60% of steroid cell tumors and less than 0.1% of all ovarian neoplasms. Steroid cell tumors (SCT) are classified depending on their cellular origin in stromal luteoma, Leydig cell tumor, and SCT-NOS. They are also divided into non-functioning and functioning, and within the last group, we can find those associated with testosterone secretion that produce virilization. Virilization signs include hirsutism, acne, low-pitched voice, androgenic alopecia, and clitoromegaly [1].

In this case report we present a 20-year-old woman with amenorrhea and virilization signs secondary to an OSCT-NOS. To our knowledge, this is the first case recorded in Mexico. The objective of this case report is to report endocrinology manifestations of a rare ovarian neoplasm and review its clinical presentation, diagnosis, treatment, and prognosis in order to alert physicians of non-common causes of virilization in young women.

## Case presentation

**Fig. 1:**
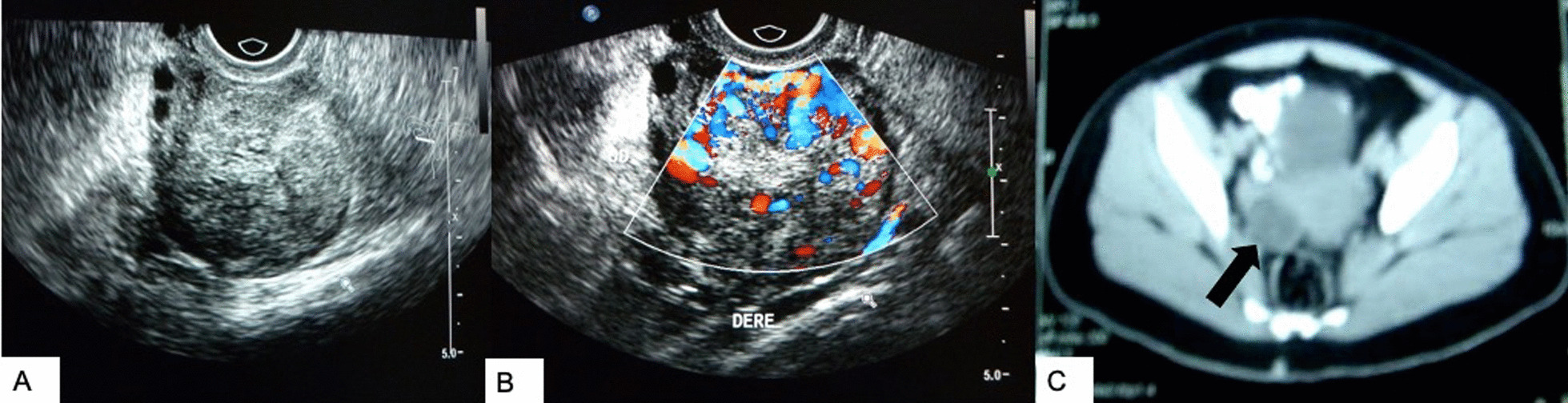
Ultrasound and CT scans of the patient. **A**, **B** Endovaginal ultrasound. We observed an enlarged right ovary due to a heterogeneous solid tumor with different echogenic areas. This area has an increased vascularity with the application of Doppler. **C** Axial slices in CT. The arrow is pointing to a hypodense solid lesion in the enlarged right ovary

A 20-year-old Mexican woman was referred to the endocrinology clinic due to secondary amenorrhea and virilization signs. She had no relevant family history or past medical history. She had thelarche, pubarche, and menarche at the age of 11, and since then she had irregular menses. At the age of 13, a diagnosis of polycystic ovarian syndrome (PCOS) was made and she was prescribed a combined oral contraceptive (OC). With this treatment her menses became regular, but 4 months after stopping this medication, her amenorrhea recurred and virilization signs worsened. At physical examination she had a heart rate of 70 beats per minute, blood pressure of 120/70 mmHg, weight of 75 kg, height of 165 cm, and a body mass index (BMI) of 27.5 kg/m^2^. She had virilization signs such as acne, low-pitched voice, androgenic alopecia in the temporal surface, hypotrophic breasts without galactorrhea, a pubic hair Tanner scale score of 5 points, breast development Tanner scale score of 3 points, clitoromegaly (clitoral index of 120 mm^2^), and a Ferriman–Gallwey score of 12 points. Laboratory tests reported a negative pregnancy test, total testosterone of 7.0 ng/mL (0.02–0.45 ng/mL), free testosterone of 47 pg/mL (0.1–6.4 pg/mL), luteinizing hormone (LH) of 2.22 mIU/mL (follicular phase 2.4–12.6 mIU/mL), follicle-stimulating hormone (FSH) of 5.27 mIU/mL (follicular phase 3.5–12.5 mIU/mL), prolactin of 4.6 ng/mL (0.6–1.2 ng/mL), 17α-hydroxy-progesterone (17-OHP) of 4.6 ng/mL (0.6–1.2 ng/mL), dehydroepiandrosterone (DEHA) of 9.5 ng/mL (1.3–9.8 ng/mL), DEHA sulfate (DHEAS) of 184 µg/dL (35–430 µg/dL), androstenedione of 24 ng/mL (0.3–3.5 ng/mL), estradiol of 32.9 pg/mL (12.5–166 pg/mL), thyroid stimulating hormone (TSH) of 2.71 mIU/mL (0.4–4.5 mIU/mL), and blood cortisol of 13 µg/dL (3.7–19.4 µg/dL). After dexamethasone suppression test, she had a 17-OHP of 2.3 ng/mL, DEHAS of 72.1 µg/dL, androstenedione of 2.59 ng/mL, and free testosterone of 13.8 pg/mL. Due to failure to normalize 17-OHP and free testosterone levels, a pelvic and transvaginal ultrasound followed by an abdominopelvic computed tomography (CT) scan were carried out, showing a right adnexal tumor (Fig. 1).

**Fig. 2:**
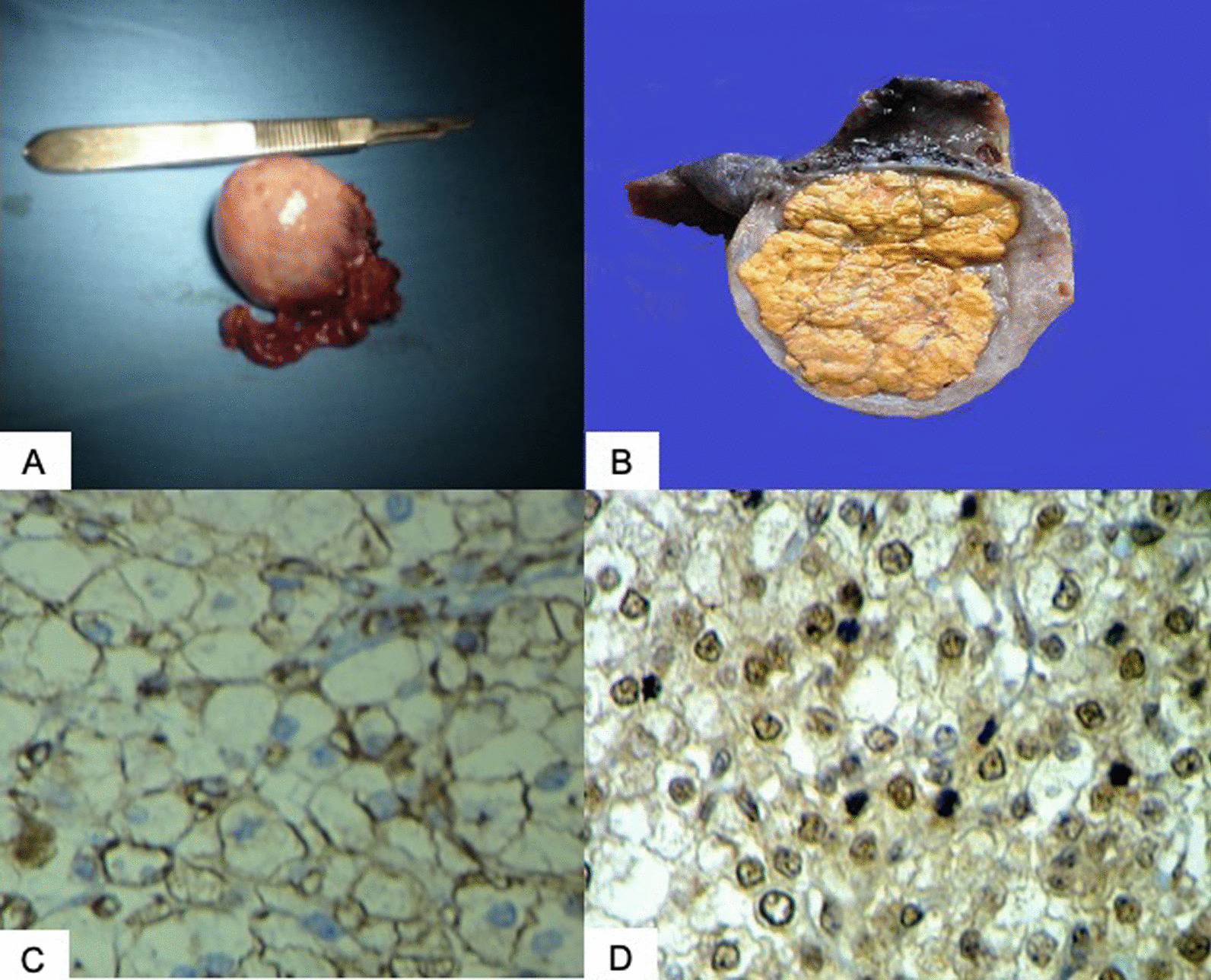
Histopathological and immunohistochemical characteristics of the tumor. **A**, **B** Resected right ovary with a size of 5 × 4 × 2 cm, with a yellow and well-circumscribed tumor inside. **C**, **D** Lipoidic cells consistent with broad eosinophilic cytoplasm with clear vacuoles inside and round nuclei with prominent nucleoli, with no Reinke crystals. We observed immunohistochemical staining was positive for vimentin (**C**) and chorionic gonadotropin hormone (**D**)

After analysis of biochemical and imaging results a multidisciplinary team decided to perform a right salpingo-oophorectomy. As seen in Fig. 2, the tumor was yellow in color, and its size was 5 × 4 × 2 cm. The final histology confirmed a tumor of lipoidic cells with large cells of broad eosinophilic cytoplasm with clear vacuoles inside and round nuclei with prominent nucleoli. No Reinke crystals were found. Immunohistochemical staining was positive for vimentin and chorionic gonadotropin hormone and negative for estrogen receptors, progesterone receptors, and androgen receptors (Fig. 2). The findings were consistent with SCT-NOS.

After surgery, her testosterone levels normalized and since then she had regular menses. A year has passed since surgery and she is currently free of disease.

## Discussion

Sex cord-stromal tumors are a rare type of ovarian tumor that account for up to 7–8% of ovarian neoplasms [2]. Within these tumors, OSCT-NOS is a very rare subtype. SCT-NOS tends to affect young women, with a mean age of 43 years and occasionally before puberty [3]. Most are benign, but 25–40% tend to have malignant activity, such as peritoneal metastasis, and they tend to be unilateral [4].

The term “steroid cell tumors” was first used by Scully in 1979, and previously they were classified as “lipid or lipoid” cell tumors. The term “not otherwise specified” implies that the cell lineage is not well defined and cannot be categorized as Leydig cell or stromal luteoma tumors [3, 5].

Its diagnosis is elusive and most of the time by exclusion. The most typical clinical manifestations of OSCT-NOS are androgen-related symptoms, such as hirsutism or virilization, present in 56–77% of cases due to most of its tumors are functioning. Furthermore, 5–6% are clinically associated with Cushing’s syndrome and 25% are non-functioning [3]. However, in case of a woman complaining of hyperandrogenic symptoms, it is important to bear in mind the wide range of differential diagnoses, such as PCOS, non-classical congenital adrenal hyperplasia, ovarian or adrenal tumors, hyperthecosis, or androgen-secreting neoplasms [2]. For this purpose, proper laboratory and imaging tests should be performed. In our case, we discarded those diagnoses due to normal DHEA and DHEAS levels, the lack of normalization of testosterone and 17-OHP levels after a suppression test with dexamethasone, the absence of polycystic ovarian morphology, and the presence of a right adnexal tumor in the ultrasonography and tomography scans. The observation that SCT had different echogenicity from the ovary and low impedance values could be useful for diagnosis [6]; however, some misdiagnoses could be reported if it has a multicystic structure [7].

The pathology characteristics of the OSCT are useful in confirming the diagnosis. Macroscopically, the SCT are often yellow due to their lipidic content, solid, and well circumscribed [8]. The evaluation of histological characteristics is mandatory for proper classification. NOS tumors lack Reinke crystals in the cytoplasm, proper of Leydig cell tumors. Additionally, they have eosinophilic cytoplasm, irregular cords, nests of large rounded-to-polygonal cells, and round nuclei with prominent nucleoli [3, 8]. Their stroma is sparse, consisting of delicate connective tissue supporting rich vascularity [8].

There are certain pathological features that indicate malignancy: two or more mitotic figures per ten high-power fields (associated with 92% of malignancy), tissular necrosis (86% of malignancy), a diameter bigger than 7 cm (78% of malignancy), hemorrhage (77% of malignancy), and nuclear atypia grade 2 or 3 (64% of malignancy) [5, 9]. In the present case, we did not find any of the characteristics mentioned above, leading us to classify it as a benign tumor. Furthermore, besides histology, in this case the immunohistochemistry revealed that tumoral cells were positive for vimentin and gonadotropin chorionic hormone and negative for estrogen receptors, progesterone receptors, and androgen receptors, confirming the diagnosis.

The primary treatment of OSCT-NOS is surgery. Other treatments, such as radiotherapy or chemotherapy, have not been tried, probably due to OSCT-NOS being rare and most often benign. There are some reports that suggest the use of gonadotropin-releasing hormone analogs (GnRHa) to decrease hormonal secretion and induce apoptosis as an attempt to avoid surgery or as a postoperative adjuvant therapy. However, the most recommended approach in young women with low-stage disease, as presented in this case, is a unilateral salpingo-oophorectomy. This surgery also allows for the preservation of fertility [3–5, 8]. However, if there is evidence of malignancy, an additional hysterectomy should be performed [2]. Most cases reported in the literature, as in this case, normalize their menstrual cycles and virilization signs along with testosterone, androstenedione, and 17-OHP levels after surgery.

## Conclusion

OSCT-NOS is a rare cause of virilization. The purpose of this case report is to alert physicians to rule out its diagnosis in young women with increased testosterone after discarding common causes such as PCOS. A multidisciplinary team including a gynecologist, endocrinologist, radiologist, and pathologist should be involved for correct diagnosis at the proper time.

## Data Availability

All relevant data are within the manuscript. More clinical information is available from the corresponding authors on reasonable request.

## Abbreviations

OSCT-NOS: Ovarian steroid cell tumors not otherwise specified
SCT-NOS: Steroid cell tumors not otherwise specified
PCOS: Polycystic ovarian syndrome
OC: Oral contraceptive
BMI: Body mass index
17-OHP: 17α-Hydroxy-progesterone
DEHA: Dehydroepiandrosterone
DHEAS: Dehydroepiandrosterone sulfate
GnRHa: Gonadotropin-releasing hormone analogs
